# Bloodstream-To-Eye Infections Are Facilitated by Outer Blood-Retinal Barrier Dysfunction

**DOI:** 10.1371/journal.pone.0154560

**Published:** 2016-05-19

**Authors:** Phillip S. Coburn, Brandt J. Wiskur, Frederick C. Miller, Austin L. LaGrow, Roger A. Astley, Michael H. Elliott, Michelle C. Callegan

**Affiliations:** 1 Department of Ophthalmology, The University of Oklahoma Health Sciences Center, Oklahoma City, Oklahoma, United States of America; 2 Oklahoma Center for Neuroscience, The University of Oklahoma Health Sciences Center, Oklahoma City, Oklahoma, United States of America; 3 Department of Cell Biology, The University of Oklahoma Health Sciences Center, Oklahoma City, Oklahoma, United States of America; 4 Department of Family and Preventative Medicine, The University of Oklahoma Health Sciences Center, Oklahoma City, Oklahoma, United States of America; 5 Department of Physiology, The University of Oklahoma Health Sciences Center, Oklahoma City, Oklahoma, United States of America; 6 Department of Microbiology and Immunology, The University of Oklahoma Health Sciences Center, Oklahoma City, Oklahoma, United States of America; Wayne State University, UNITED STATES

## Abstract

The blood-retinal barrier (BRB) functions to maintain the immune privilege of the eye, which is necessary for normal vision. The outer BRB is formed by tightly-associated retinal pigment epithelial (RPE) cells which limit transport within the retinal environment, maintaining retinal function and viability. Retinal microvascular complications and RPE dysfunction resulting from diabetes and diabetic retinopathy cause permeability changes in the BRB that compromise barrier function. Diabetes is the major predisposing condition underlying endogenous bacterial endophthalmitis (EBE), a blinding intraocular infection resulting from bacterial invasion of the eye from the bloodstream. However, significant numbers of EBE cases occur in non-diabetics. In this work, we hypothesized that dysfunction of the outer BRB may be associated with EBE development. To disrupt the RPE component of the outer BRB *in vivo*, sodium iodate (NaIO_3_) was administered to C57BL/6J mice. NaIO_3_-treated and untreated mice were intravenously injected with 10^8^ colony forming units (cfu) of *Staphylococcus aureus* or *Klebsiella pneumoniae*. At 4 and 6 days postinfection, EBE was observed in NaIO_3_-treated mice after infection with *K*. *pneumoniae* and *S*. *aureu*s, although the incidence was higher following *S*. *aureus* infection. Invasion of the eye was observed in control mice following *S*. *aureu*s infection, but not in control mice following *K*. *pneumoniae* infection. Immunohistochemistry and FITC-dextran conjugate transmigration assays of human RPE barriers after infection with an exoprotein-deficient *agr/sar* mutant of *S*. *aureus* suggested that *S*. *aureus* exoproteins may be required for the loss of the tight junction protein, ZO-1, and for permeability of this *in vitro* barrier. Our results support the clinical findings that for both pathogens, complications which result in BRB permeability increase the likelihood of bacterial transmigration from the bloodstream into the eye. For *S*. *aureus*, however, BRB permeability is not required for the development of EBE, but toxin production may facilitate EBE pathogenesis.

## Introduction

The blood-retinal barrier (BRB) is a component of ocular immune privilege and serves to protect the delicate, nonregenerative neural retina from the immune system and bloodborne pathogens. The BRB consists of inner (endothelial cells, pericytes, and astrocytes) and outer (retinal pigment epithelial cells) components. The retinal pigment epithelium (RPE) consists of a single layer of cuboidal pigmented cells whose specific functions are critical for neural retina homeostasis. The RPE maintains the retinal environment by limiting transport across the retina, thus, maintaining a tight barrier to choroidal bloodborne substances [1,2]. The endothelial cells lining the capillaries supplying the retina with oxygen and nutrients form the inner BRB, which exhibits selective permeability to small molecules, and is virtually impermeable to large macromolecules [3]. During the development of diabetes and its ocular complication diabetic retinopathy, changes occur in the BRB which result in greater vascular permeability and loss of RPE function [4–20].

Diabetes is the leading predisposing condition for the development of endogenous bacterial endophthalmitis (EBE) [21], a severe, often blinding intraocular infection emanating from the bloodstream [21–27]. In 60% of cases of EBE, an underlying condition is present, and diabetes is present in 33% of those cases [21]. EBE occurs at a frequency of approximately 2% to 8% of all cases of endophthalmitis. Patients with EBE typically present with ocular pain, blurring or loss of vision, a hypopyon, an insufficient fundus view, and photophobia. Infection of the eye via this route can result in vision loss, and in the worst-case scenario, enucleation or evisceration of the globe. EBE can also affect both eyes at the same time, causing bilateral blindness. Jackson *et al*. [21] reported in a recent review of 342 EBE cases from 1986 to 2012 that the median final visual acuity after EBE was 20/100. In 44% of these cases, visual acuities were worse than 20/200. In approximately 24% of all cases examined, patients required evisceration or enucleation of the globe. Associated mortality in these EBE cases was 4% [21]. The leading causes of Gram-negative and Gram-positive EBE are *Klebsiella pneumoniae* and *Staphylococcus aureus*, respectively [21–27]. Despite antibiotic and surgical intervention, the clinical outcome for patients with EBE continues to be poor [21].

Our previous studies suggest that during diabetes, a compromised BRB serves as a portal for bacteria to gain access to the eye from the bloodstream [28,29]. We reported that an increased incidence of *K*. *pneumonaie* and *S*. *aureus* EBE in a diabetic murine model correlated with the length of time following diabetes induction with streptozotocin (STZ) [28,29]. This increased EBE incidence also correlated with greater vascular permeability in the eyes of STZ-induced diabetic mice [28,29]. Our results supported clinical reports that diabetes is a predisposing risk factor for the development of EBE [28,29]. However, diabetes progression results in a myriad of other host changes, including immunological deficits such as the inability of inflammatory cells to phagocytize *K*. *pneumoniae* and *S*. *aureus* [30,31]. To dissect the specific mechanisms that underlie EBE development, we sought to divorce BRB permeability from the immunological changes that occur during diabetes progression. Specifically, we hypothesized that dysfunction of the RPE, a component of the outer BRB which is altered during the development of diabetes, facilitates the development of EBE. To test this hypothesis, we selectively induced RPE degeneration using sodium iodate (NaIO_3_), an oxidizing agent that exerts toxicity specifically towards the RPE [32] and is a neurodegenerative insult [33]. In the present study, EBE incidence after infection with *S*. *aureus* and *K*. *pneumoniae* in NaIO_3_-treated mice was comparable to the incidence observed in the diabetic EBE models [28,29]. In control mice, *S*. *aureus* infection resulted in EBE, but *K*. *pneumoniae* infection did not. Furthermore, we observed that *S*. *aureus* exoprotein production was associated with a disruption in ZO-1 staining and increased permeability of an *in vitro* RPE barrier. Our results therefore suggest that alterations in the RPE component of the outer BRB may serve as a mechanism by which *K*. *pneumoniae* and *S*. *aureus* EBE develops, but these alterations are not required for *S*. *aureus* EBE to occur.

## Results

### Permeabilization of the RPE with sodium iodate

**Fig 1:**
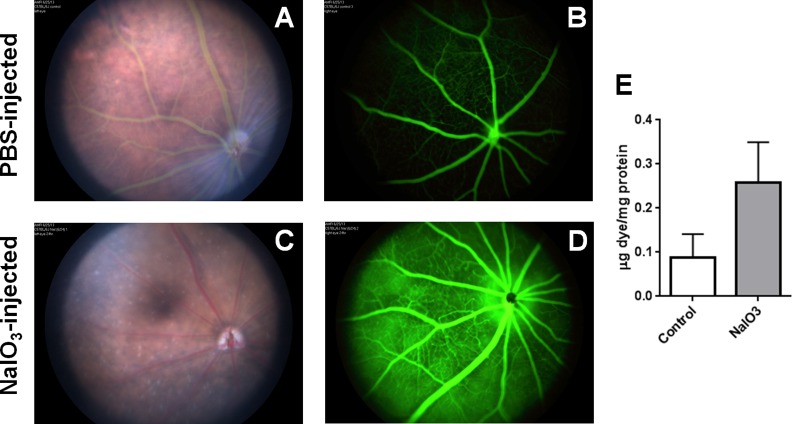
Blood-retinal Barrier Breakdown in NaIO_3_-treated mice. **(A-D)** Funduscopic imaging of mouse eyes 24 hours after injection of either PBS or NaIO_3_. In PBS-injected mice (A and B), the fluorescence from the AK-FLUOR dye demarcates the retinal and/or choroidal vasculature and distinguishes it from adjacent areas/structures. In NaIO_3_-injected mice (C and D), note the diffuse fluorescence resulting from increased outer BRB permeability and leakage of the dye. **(E)** Albumin leakage into the retina after injection of either PBS or NaIO_3_ was quantified using a modified Evans Blue protocol. Bars represent mean ± standard deviation (SD) for N ≥ 5 animals for all groups. A two-tailed t-test was used to assess significance between PBS-injected and NaIO_3_-injected mice (P = 0.01).

To determine whether alterations specifically in the RPE resulted in an increased incidence of EBE, we first disrupted the RPE of C57BL/6J mice with sodium iodate (NaIO_3_) [1,2,32]. The extent of damage to and resulting permeability of the RPE barrier was visualized *in vivo* by fluorescein angiography. After 24 hours, significant changes in RPE pigmentation were observed in NaIO_3_-treated mice (Fig 1C), but not in PBS-treated mice (Fig 1A). The retinal vasculature and optic nerve tissue in these eyes appeared normal. Wang *et al*. observed similar effects in retinal tissue following treatment of C57BL/6J mice with 20 and 30 mg/kg NaIO_3_ from 1 to 8 days after injection [32]. NaIO_3_ treatment resulted in extensive leakage of fluorescein dye into the vitreous relative to PBS-injected mice. In PBS-treated mice, the fluorescence from the AK-FLUOR dye (Fig 1B) demarcated the retinal and/or choroidal vasculature, distinguishing it from adjacent areas and structures. In Fig 1D, diffuse fluorescence resulting from increased permeability and leakage of the dye was observed in NaIO_3_-treated mice.

To quantify the extent of permeability of the RPE in NaIO_3_-treated and untreated mice, a modified Evans Blue dye assay was employed to measure albumin leakage into the retina [34]. Eyes from mice treated with sodium iodate allowed a greater concentration of albumin into the retina compared to that of untreated mice (Fig 1E, P = 0.01). Together, these results demonstrated that NaIO_3_ disrupted the barrier properties of the RPE and rendered mouse eyes permeable to AK-FLUOR and albumin 24 hours following treatment.

### RPE dysfunction and incidence of *S*. *aureus* and *K*. *pneumoniae* EBE

To establish a link between RPE dysfunction and the development of EBE, groups of mice were infected 24 hours after intraperitoneal injection of either PBS or NaIO_3_. These data are summarized in Table 1. At 96 hours postinfection with *K*. *pneumoniae*, 3 out of 10 NaIO_3_-treated mice developed EBE. One eye from each mouse was infected. The mean *K*. *pneumoniae* cfu per eye among the NaIO_3_-treated mice was 3.04 x 10^2^. None of the control mice infected with *K*. *pneumoniae* developed EBE. At 96 hours postinfection with *S*. *aureus*, 6 out of 10 NaIO_3_-treated mice (6 eyes) developed EBE and 2 out of 10 control mice (2 eyes) developed EBE. The mean *S*. *aureus* cfu per eye was 2.72×10^2^ for the NaIO_3_-treated mice, and 2.48×10^2^ for the control mice. NaIO_3_-induced RPE permeabilization resulted in a 30% *K*. *pneumoniae* EBE incidence, similar to the 27% *K*. *pneumoniae* EBE incidence that we previously reported for mice rendered diabetic for 5 months [28]. NaIO_3_-induced RPE permeabilization also resulted in a 60% *S*. *aureus* EBE incidence, similar to the 58% *S*. *aureus* EBE incidence we observed in our 3-month diabetic mice [29].

**Table 1:** Incidence of *K*. *pneumoniae* and *S*. *aureus* EBE at 4 days postinfection in control and NaIO_3_-treated mice.

	Control,*K*. *pneumoniae infected*	NaIO_3_ treated,*K*. *pneumoniae* infected	Control,*S*. *aureus infected*	NaIO_3_ treated,*S*. *aureus* infected
Number of mice infected	10	10	10	10
Deaths during infection course	0	0	0	0
Number euthanized prior to 96 hours post-infection	0	0	0	0
Number surviving after 96 hours post-infection	10	10	10	10
Mice with EBE	0	3	2	6
% Infected of Survivors	0	30	20	60
Mean CFU/eye	0	3.04 x 10^2^	2.48×10^2^	2.72×10^2^
Standard Deviation	0	(±3.42×10^2^)	(±2.77×10^2^)	(±3.12×10^2^)

Assessment of EBE incidence in NaIO_3_-treated mice 6 days after infection with both pathogens (Table 2) revealed a 20% incidence of *K*. *pneumoniae* EBE and a 50% incidence of *S*. *aureus* EBE, but no infections in the control mice. After 6 days postinfection, the mean cfu per eye for the *K*. *pneumoniae*-infected mice was 1.16 x 10^2^, and for *S*. *aureu*s-infected mice was 2.58×10^2^. These results indicated that intraocular infection with either *K*. *pneumoniae* or *S*. *aureus* can occur after specific disruption of the RPE component of the outer BRB in nondiabetic mice at incidences similar to that reported in diabetic mice [28,29]. Similar to what we observed previously [29], *S*. *aureus* caused EBE even when the BRB was intact in control mice not treated with NaIO_3_, while *K*. *pneumoniae* did not cause infections in these mice.

**Table 2:** Incidence of *K*. *pneumoniae* and *S*. *aureus* EBE at 6 days postinfection in control and NaIO_3_-treated mice.

	Control,*K*. *pneumoniae infected*	NaIO_3_ treated,*K*. *pneumoniae* infected	Control,*S*. *aureus infected*	NaIO_3_ treated,*S*. *aureus* infected
Number of mice infected	5	5	5	5
Deaths during infection course	0	0	0	1
Number euthanized prior to 96 hours post-infection	0	0	0	0
Number surviving after 96 hours post-infection	5	5	5	4
Mice with EBE	0	1	0	2
% Infected of Survivors	0	20	0	50
Mean CFU/eye	0	1.16 x 10^2^	0	2.58×10^2^
Standard Deviation	0	0	0	(±14)

Wang *et al*. reported significant effects of NaIO_3_ treatment on the scotopic and photopic b-wave ERG responses, showing almost complete elimination of ERG responses at 8 days following treatment [32]. In our previous study [29], we reported no changes in ERG responses 4 days following infection with *S*. *aureus* in diabetic animals, likely due to the low numbers of bacteria detected in those infected eyes [28,29]. In the current study, ERGs were not performed on infected mice because of the anticipated low numbers of bacteria in these eyes and because the interpretation of any observed ERG decrease could be potentially confounded by the effects of NaIO_3_ treatment.

### *S*. *aureus*-induced alterations in an *in vitro* human outer BRB are exoprotein-dependent

**Fig 2:**
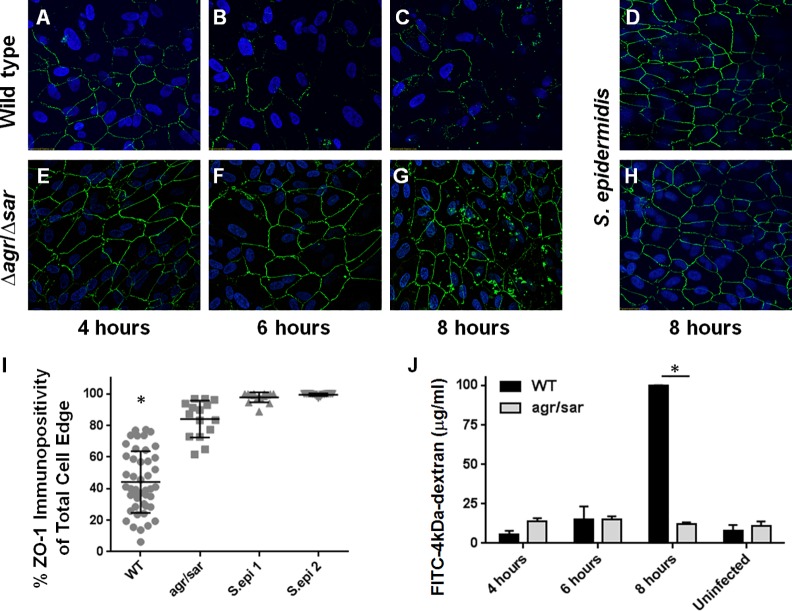
Exoprotein-dependent alterations in ZO-1 immunoreactivity of cultured human RPE cells infected with *S*. *aureus*. **(A-H)** Human ARPE-19 monolayers were infected with wild type *S*. *aureus* 8325–4 (A-C), an *agr/sar*-deficient mutant (E-G), or two ocular isolates of *S*. *epidermidis* (D and H), each at a concentration of 10^4^ cfu/ml, MOI = 0.02. After 4, 6, or 8 hours postinfection, monolayers were stained with anti-ZO-1 and analyzed by immunofluorescence microscopy (10x magnification). **(I)** Quantitative analysis of ZO-1 staining demonstrates the exoprotein-dependency of ZO-1 disruption during *S*. *aureus* infection. The y-axes represent percent immunopositivity for anti-ZO-1 from 5 randomly-selected cells from each of N≥10 separate fields (*S*. *aureus* 8325–4 infected RPE cells versus *S*. *aureus* RN6390 *agr/sar* infected at 8 hours postinfection, *P<0.0001). **(J)** Alterations in the permeability of a cultured RPE barrier are dependent on *S*. *aureus* exoprotein production. Intact monolayers of human RPE cells in 0.4 micron transwells were infected with *S*. *aureus* 8325–4 or RN6390 *agr/sar* at a concentration of 10^4^ cfu/ml (MOI = 0.01). After 4–8 hours of infection, diffusion of FITC-4-kDa-dextran across the monolayer was assessed by fluorescence spectrometry of media from the bottom chamber. After 8 hours, the fluorescence intensity in the bottom chamber media was significantly greater after infection with 8325–4 than after infection with the *agr/sar*-deficient strain (*P<0.0001). Values represent the mean concentration of the conjugate in the bottom chamber ± the SD (N≥3 at each time point) based on extrapolation from a standard curve of the fluorimetry of known FITC-dextran concentrations.

We previously reported that *S*. *aureus*, but not *K*. *pneumoniae*, caused a significant reduction in immunoreactivity of the tight junction protein ZO-1 between cultured human RPE cells in our *in vitro* model of the outer BRB, suggesting that *S*. *aureus* is able to directly disrupt the expression and/or organization of tight junctions between RPE cells [29]. Disruption of ZO-1 immunostaining correlated with changes in the permeability of our *in vitro* outer BRB model to both FITC-dextran conjugate molecules and to live *S*. *aureus* [29]. Because RPE dysfunction was necessary for invasion of *K*. *pneumoniae* but not *S*. *aureus* into the eye from the bloodstream, the question arose whether RPE alterations were the direct result of *S*. *aureus* exoprotein production. We therefore examined the ability of an *agr/sar* quorum sensing-deficient, exoprotein-deficient mutant of *S*. *aureus* [35] to alter ZO-1 immunoreactivity. Immunofluorescence microscopy revealed that infection with wild type *S*. *aureus* caused progressive disruption in ZO-1 staining over time (Fig 2, panels A-C), while infection with the *agr/sar*-deficient mutant (Fig 2, panels E-G) or two ocular isolates of *S*. *epidermidis* (Fig 2, panels D and H) did not. The percent of ZO-1 immunopositivity of RPE monolayers infected with wild type *S*. *aureus* was significantly less than in RPE monolayers infected with the *agr/sar*-deficient mutant or two ocular isolates of *S*. *epidermidis* (P<0.0001, Fig 2, panel I). These results suggested that the observed changes in ZO-1 immunostaining after infection with wild type *S*. *aureus* were dependent on exoprotein production. RPE viability was greater than 95% at all time points, as determined by trypan blue staining [29].

Intact monolayers of human RPE cells in 0.4 micron transwells were infected with wild type *S*. *aureus* or the *agr/sar*-deficient mutant and diffusion of FITC-4-kDa-dextran across the barrier and into the bottom chamber was assessed by fluorescence spectrometry (Fig 2, panel J). No significant differences in RPE monolayer permeability were observed after 4 or 6 hours of infection with the two strains. However, after 8 hours, the fluorescence intensity was significantly greater in the bottom chamber of RPE monolayers infected with wild type *S*. *aureus* (p<0.0001) compared with that of RPE monolayers infected with the exoprotein-deficient mutant. These results indicated that *S*. *aureus* could disrupt ZO-1 and the permeability of our *in vitro* human outer BRB in an exoprotein-dependent manner. Taken together, these findings substantiate our observation that the greater incidence of *S*. *aureus* EBE compared to *K*. *pneumoniae* EBE correlates with the capacity of *S*. *aureus* to directly disrupt RPE tight junctions, and suggests that *S*. *aureus* migration across the outer BRB may be facilitated by its toxic exoproteins.

## Discussion

Endogenous bacterial endophthalmitis is an often devastating bacterial infection of the eye originating from remote sites in the body. This disease is associated with a number of underlying conditions, but diabetes is a frequent risk factor [21]. During the development of diabetic retinopathy, there is degeneration of and an increase in the permeability of the BRB. We previously reported that the environment created by a compromised BRB promoted the entry of bloodborne pathogens into the eye. In mice with STZ-induced diabetes, we previously observed 24% (3-month) and 27% (5-month) incidences of *K*. *pneumoniae* EBE [28]. We also observed *S*. *aureus* EBE among 3-month (58% incidence) and 5-month (33% incidence) diabetic mice. Although the frequency of *S*. *aureus* EBE in 5-month diabetic mice was comparable to previous observations for *K*. *pneumoniae* EBE, the incidence of *S*. *aureus* EBE in 3-month diabetic mice was 2.5-fold greater. We found no *K*. *pneumoniae* EBE in control (nondiabetic) or 1-month diabetic mice, but observed a 7% and a 12% incidence of *S*. *aureus* EBE in these same groups, respectively. These data implied that *S*. *aureus* was capable of invading the eye regardless of the degree of BRB integrity, and raised the possibility that *S*. *aureus* might directly affect the outer BRB, resulting in infection of the eye. Jung *et al*. recently reported that 9% of *S*. *aureu*s bacteremia patients developed ocular infections [36], but only 30% of those had diabetes as an underlying condition [36]. The *S*. *aureus* EBE cases in that study were primarily associated with infective endocarditis, providing clinical support for our hypothesis that *S*. *aureus* can cross the BRB and invade the eye in the absence of diabetes-related changes to the BRB. Our hypothesis is also supported by findings in a murine model of hematogenous *S*. *aureus* meningitis [37]. Sheen *et al*. reported that *S*. *aureus* was capable of crossing the blood-brain barrier (BBB) and infecting the brains of normal CD-1 mice [37]. After tail vein injection of 2 x 10^8^ cfu of *S*. *aureus*, bacteria were detected in the brains of 7 out of 9 infected animals at concentrations ranging from approximately 10^2^ to greater than 10^6^ cfu per gram of brain tissue at 96 hours postinfection [37]. Although no eyes were analyzed in that study, these findings demonstrate that *S*. *aureus* can infiltrate intact barriers of the central nervous system.

NaIO_3_ treatment has been utilized as a model for RPE degeneration of the retina [38]. NaIO_3_ may increase the ability of melanin to convert glycine into toxic glucoxylate. NaIO_3_ also inhibits the activities of various enzymes which contribute to cellular energy production (triose phosphate, lactate, and succinyl dehydrogenases). [1,2]. NaIO_3_ has been shown to exert toxicity to the RPE in a number of mammalian species, including mice [39]. Toxicity to other organs or tissues has not been observed [2,32]. NaIO_3_ injection of 20 to 30 mg/kg in C57BL6/J mice caused loss of retinal pigmentation and atrophy as early as 3 days after treatment [32]. Histological analysis revealed swelling of the RPE and migration of pigmented cells into the outer segment during this time frame. No acute inflammation was reported [32]. Immunostaining revealed loss of RPE65 8 days following treatment and reductions in scotopic and photopic b-wave amplitudes that reached zero by day 8 [32]. These results showed that functional deficits occurred as early as 1 day post-NaIO_3_ treatment, with significant morphological changes occurring thereafter. Based on these results, we chose a concentration of 50 mg/kg of NaIO_3_ and infection at 24 hours after treatment as a sufficient dosage and length of time to affect functional changes in the RPE.

In the current study, we established that direct disruption of the RPE component of the outer BRB by NaIO_3_ led to increased RPE permeability and an increase in EBE incidence with *K*. *pneumoniae* and *S*. *aureus*. The incidence of EBE due to each pathogen after NaIO_3_ treatment was similar to what we observed in our diabetic mouse model, suggesting that disruption of the RPE barrier facilitated the initiation and development of EBE. Our current results suggest that an intact and functional RPE is critical for preventing infection with *K*. *pneumoniae*, as evidenced by the lack of infection in control nondiabetic mice [28] and control mice not treated with NaIO_3_. In contrast, our observations of *S*. *aureus* EBE in control nondiabetic and untreated animals, albeit at a lower frequency than in diabetic and treated animals, suggests that *S*. *aureus* is able induce outer BRB barrier dysfunction on its own.

In addition to its barrier function [40], the RPE provides the retina with a number of essential functions, including nutrient transport and waste removal, regeneration of the visual pigment, and removal of photoreceptor outer segments. The RPE contributes to the normal immune privilege of the eye and restricts bloodstream access to the sensitive neuroretina. Pitkanen *et al*. conducted a systematic study of the permeability of isolated bovine RPE [40]. This group showed that the bovine RPE-choroid was 10 to 100 times less permeable to a series of fluorescent probes of differing molecular masses (ranging from 376 to 77,000 Da) than the sclera, and that the permeability of the RPE exponentially decreased with an increase in the molecular radius of the fluorescent compounds [40]. These experiments demonstrated that the RPE functions as a major permeability barrier to the choroidal vasculature due to the intracellular tight junctions.

The RPE also shields the sensitive neural retina from pathogens circulating in the bloodstream, and therefore disruption of the RPE could create a portal for bacteria in the fenestrated choroidal capillaries to enter the eye. We previously reported a decrease in ZO-1 immunoreactivity at the RPE borders of an *in vitro* human model of the outer BRB after infection with *S*. *aureus*, but not after *K*. *pneumoniae* infection [29]. Disruption of ZO-1 immunostaining correlated with a decrease in RPE barrier function, as measured by increases in FITC-conjugated dextran permeability and *S*. *aureus* transmigration across the barrier at 6 and 8 hours following *S*. *aureus* infection. These results supported our *in vivo* findings with *K*. *pneumoniae* and *S*. *aureus* EBE, and suggested that *S*. *aureus* directly contributed to the development of EBE by disrupting RPE barrier function. In the current study, infection of the *in vitro* human outer BRB model with an exoprotein-deficient mutant of *S*. *aureus* [35] resulted in significantly less ZO-1 alteration relative to a toxigenic *S*. *aureus* strain, suggesting a possible role for toxic exoproteins in altering the outer BRB. These results were similar to the toxin-dependent disruption of an *in vitro* RPE barrier by *B*. *cereus* [41]. *S*. *aureus* elaborates a number of exoproteins that are regulated by the *agr* quorum sensing system and include the α-, β-, γ-, and δ-toxins, the Panton-Valentine leukocidin (PVL), enterotoxins B-D, exfoliative toxins A and B, toxic-shock syndrome toxin-1, V8 protease, serine and cysteine proteases, phospholipase, staphylokinase, and hyaluronidases [42–47, 48]. The *sar*-regulated factors include the δ-toxin, coagulase, and the surface fibronectin binding proteins A and B [48]. Previous analysis of experimental exogenous endophthalmitis initiated by toxin-deficient *S*. *aureus* demonstrated that toxin production is very important to pathogenesis [49–51]. These toxins may directly damage intraocular tissues and may factor into EBE pathogenesis by interacting with and disrupting the RPE barrier, resulting in *S*. *aureus* invasion into the retinal vasculature. However, enterotoxin A, that is regulated independently of *agr*, and enterotoxins B-D that can be elaborated at higher levels independently of the *agr* system [59] could all potentially contribute to this process. Sheen *et al*. [37] reported a correlation between *S*. *aureus* invasion across an *in vitro* BBB model of human brain microvascular endothelial cells and the presence of cell-associated liptotechoic acid (LTA) [37]. Deletion of *ypfP*, the gene encoding the glycosyltransferase responsible for synthesizing the glycolipid moiety that anchors LTA to the cytoplasmic membrane, resulted in decreased invasion in the *in vitro* BBB model and infection in the mouse meningitis model [37]. These results suggested that *S*. *aureus* might utilize factors other than toxins to invade the central nervous system or, in our case, the eye via the outer BRB.

In summary, our models support the clinical findings that for both pathogens, complications which result in BRB permeability increase the likelihood of transmigration of *K*. *pneumoniae* and *S*. *aureus* from the bloodstream into the eye. RPE compromise is a key element of EBE pathogenesis in this model, but it is clear that the mechanisms by which different pathogens cause EBE are unique to each species. Identifying the critical host and pathogen factors that contribute to this blinding infection is critical when devising improved therapeutic strategies for treating a disease that has experienced only incremental therapeutic success over several decades.

## Materials and Methods

### Animals and Ethics Statement

This study was carried out in strict accordance with the recommendations in the Guide for the Care and Use of Laboratory Animals of the National Institutes of Health. The protocol was approved by the Institutional Animal Care and Use Committee of the University of Oklahoma Health Sciences Center (Protocol numbers 12–100 and 13–086). Six week old C57BL/6J mice were acquired from the Jackson Laboratory (Catalog 000664, Bar Harbor ME). Mice were allowed to adjust to conventional housing two weeks prior to PBS/NaIO_3_ injection. Mice were anesthetized with a cocktail of 85 mg ketamine/kg and 14 mg xylazine/kg prior to tail-vein injections of bacteria.

### RPE Permeabilization

Male C57BL/6J mice were intraperitoneally injected with sodium iodate (NaIO_3_, 50 mg/kg) to induce RPE permeabilization [1,2,32]. Controls consisted of mice intraperitoneally injected with PBS (pH 7.4).

### Fluorescent Angiography

Male C57BL/6J control and NaIO_3_-injected mice were imaged at 24 hours postinjection using a Micron III Retinal Imaging System (Phoenix Research Laboratories, Inc., Pleasanton, CA). Following general anesthesia, 0.002 mL of AK-FLUOR® 10% (100mg/mL) was injected intraperitoneally two minutes prior to fluorescein angiography [1,2,32].

### Evans Blue Dye Vascular Permeability Assay

Albumin leakage from blood vessels into the retina was quantified using a modified Evans Blue protocol [34]. NaIO_3_-treated or PBS-treated mice were anesthetized and 15 mg Evans Blue dye (Sigma-Aldrich, St. Louis, MO) per kg was intraperitoneally injected. The Evans Blue dye leakage assay was performed as previously described [28]. The OD_620_ of the supernatants was measured and the concentration of Evans Blue was calculated from a standard curve. Pellets were then solubilized in 0.2% SDS in PBS and protein concentrations measured using a BCA protein assay. The concentration of Evans Blue in each sample was then normalized to the total protein per sample. Results were expressed in micrograms of Evans Blue/mg total protein content.

### Endogenous Bacterial Endophthalmitis (EBE) Model

A hypermucoviscosity (HMV)-negative *K*. *pneumoniae* endophthalmitis isolate (KLP02) [52] and *S*. *aureus* strain 8325–4, a well-characterized prophage and plasmid-free strain derived from the clinical ocular isolate 8325 [53], were utilized for our studies [28,29,52]. Both strains were grown for 18 hours in brain heart infusion media (BHI; Difco Laboratories, Detroit, MI) and subcultured in pre-warmed BHI to logarithmic phase. Bacteria were then centrifuged and resuspended in phosphate buffered saline (PBS). EBE was established by injecting mice via the tail vein with 10^8^ colony forming units (cfu) in 100 μl PBS, as previously described [28,29]. At 4 and 6 days postinfection, both eyes from each mouse were harvested for bacterial quantitation.

### Bacterial Quantitation

Both eyes from each mouse were enucleated, placed into separate tubes of sterile PBS and 1.0 mm sterile glass beads and homogenized for 60 seconds at 5,000 rpm in a Mini-BeadBeater (Biospec Products, Inc., Bartlesville, OK). Eye homogenates were serially diluted and plated in triplicate on BHI agar plates for *K*. *pneumoniae*-infected mice, and tryptic soy agar (TSA) + 5% sheep erythrocyte and mannitol salt agar plates for *S*. *aureus*-infected mice. After overnight incubation at 37°C, the cfu per eye was determined as previously described [28,29,54,55].

### *In Vitro* Human Outer BRB Model

The *in vitro* human outer BRB model was established as previously described using human ARPE-19 cells (CRL-2302, American Type Culture Collection, Manassas, VA) propagated and maintained in Dulbecco modified Eagle medium (DMEM)/F12 (Life Technologies, Grand Island, NY) supplemented with 10% fetal bovine serum (FBS, Life Technologies) [29]. *S*. *aureus* strain 8325–4 and RN6390 *agr/sar* [49, 53, 35] were grown for 18 hours in BHI medium, washed with PBS, and diluted into RPE cell culture medium. The parental strain RN6390 is a direct descendant of 8325–4 [56] and the *agr/sar*-deficient mutant has been used to initiate endophthalmitis in rabbits [49]. The ocular virulence of this quorum sensing-deficient double mutant was significantly less than that of wild type *S*. *aureus* [49]. Tissue culture wells containing either glass coverslips or transwells with confluent monolayers of human ARPE-19 cells were inoculated with the bacterial suspension to achieve a final concentration of 10^4^ cfu/ml. This represented a multiplicity of infection (MOI) of 0.02, or 1 bacterial cell per 50 RPE cells on the coverslips, and an MOI of 0.01, or 1 bacterial cell per 100 RPE in the transwells. Following infection, bacterial growth was assessed at 2 hour intervals. Mock, uninfected coverslips or transwells were incubated with RPE cell culture medium only.

### Immunocytochemistry

Infected RPE monolayers on coverslips were fixed in 100% methanol at -80°C for 30 minutes. Coverslips were incubated once in TBS + 0.25% Triton-X100 for 10 minutes, followed by Protein Block (DakoCytomation, Carpinteria CA) for 10 minutes at room temperature. Anti-ZO-1 antibody (Invitrogen, Carlsbad CA) was added to a final concentration of 15 μg/mL. The anti-ZO-1 antibody was removed and the coverslips were washed 3 times with PBS + 0.001% Tween 20. Alexa Fluor 488 goat anti-mouse IgG (1:200 dilution) (Life Technologies, Eugene, OR) was added and coverslips were incubated for 30 minutes at room temperature. After 3 washes with PBS + 0.001% Tween 20, coverslips were mounted on glass slides with Vectashield Hard Set, with DAPI (Vector, Burlingame, CA), and imaged by confocal microscopy (Olympus Confocal FV500, Waltham, MA). The fluorescence intensity of ZO-1 staining at the periphery of individual RPE cells was quantified using Image J [29,57–59]. Briefly, N≥10 random confocal images were taken per group and N = 5 cells were chosen at random from each confocal image. The edge of each cell was traced and a plot profile of intensity for each trace was generated (approximately 500–1000 points per trace). The percent ZO-1 immunopositivity for each cell was calculated as the fraction of points greater than 25% of the maximum intensity for each cell. Percentages for each group were averaged and are presented as the mean ± SD for N≥10 images per group (*S*. *aureus* 8325–4, *S*. *aureus* RN6390 *agr/sar* and two *Staphylococcus epidermidis* ocular isolates [negative controls at equivalent concentrations] at 8 hours postinfection).

### FITC-Dextran Conjugate Diffusion Assay

To measure the degree of permeability of human RPE cell culture monolayers after infection with *S*. *aureus* 8325–4 and RN6390 *agr/sar*, the diffusion of a 4 kDa fluorescein isothiocyanate (FITC)-dextran conjugate across the monolayer was assessed by fluorescent spectrophotometry as previously described [29]. Monolayers were cultured on 0.4 μm transwells and infected with 10^4^ cfu/mL in RPE cell culture medium or medium alone for 4, 6, or 8 hours postinfection. Addition of hydrogen peroxide (H_2_O_2_, Sigma-Aldrich) to a final concentration of 30% for 30 minutes served to permeabilize the monolayers and functioned as a positive control. The 4 kDa FITC-dextran conjugate at 1 mg/mL were added to the transwells at each time point and incubated for 1 h at 37°C. Fluorescence was measured in the lower chamber by fluorescence spectroscopy, and the concentration of the 4 kDa FITC-dextran conjugate that diffused across the monolayer was calculated from a standard curve of known concentrations. Values are expressed as the mean FITC-dextran conjugate concentration ± SD of N = 3 measurements per time point.

### Statistics

All values represent the mean ± standard deviation (SD) of the bacterial counts in infected eyes, Evans Blue dye values, and FITC-dextran conjugate concentrations. Two-tailed, 2-sample t-tests were used for statistical comparisons between groups for the Evans Blue dye leakage and FITC-dextran conjugate diffusion assays. The Mann-Whitney U test was used to assess levels of significance in the ZO-1 immunopositivity assay. A P value of <0.05 was considered significant.

## References

[1] Enzmann V, Row BW, Yamauchi Y, Kheirandish L, Gozal D, Kaplan HJ, McCall MA. Behavioral and anatomical abnormalities in a sodium iodate-induced model of retinal pigment epithelium degeneration. Exp Eye Res. 2006;82: 441–448. 16171805 10.1016/j.exer.2005.08.002

[2] Kiuchi K, Yoshizawa K, Shikata N, Moriguchi K, Tsubura A. Morphologic characteristics of retinal degeneration induced by sodium iodate in mice. Curr Eye Res. 2002;25: 373–379. 12789545 10.1076/ceyr.25.6.373.14227

[3] Campbell M, Humphries P. The blood-retina barrier: tight junctions and barrier modulation. Adv Exp Med Biol. 2012;763: 70–84. 23397619

[4] Schroder S, Palinski W, Schmid-Schonbein GW. Activated monocytes and granulocytes, capillary nonperfusion, and neovascularization in diabetic retinopathy. Am J Pathol. 1991;139: 81–100. 1713023 PMC1886150

[5] Miyamoto K, Hiroshiba N, Tsujikawa A, Ogura Y. *In vivo* demonstration of increased leukocyte entrapment in retinal microcirculation of diabetic rats. Invest Opthalmol Vis Sci. 1998;39: 2190–2194. 9761301

[6] Miyamoto K, Khosrof S, Bursell S–E, et al Prevention of leukostasis and vascular leakage in streptozotocin-induced diabetic retinopathy via intercellular adhesion molecule-1 inhibition. Proc Natl Acad Sci USA. 1999;96: 10836–10841. 10485912 10.1073/pnas.96.19.10836 PMC17969

[7] Funatsu H, Yamashita H, Sakata K, Noma H, Mimura T, Suzuki M, et al Vitreous levels of vascular endothelial growth factor and intercellular adhesion molecule 1 are related to diabetic macular edema. Ophthalmology. 2005;112: 806–816. 15878060 10.1016/j.ophtha.2004.11.045

[8] Fong DS, Aiello L, Gardner TW, King GL, Blankenship G, Cavallerano JD, Ferris FL 3rd, Klein R. American Diabetes Association: Diabetic retinopathy. Diabetes Care. 2003;26: 226–229. 12502685 10.2337/diacare.26.1.226

[9] Neely KA, Gardner TW. Ocular neovascularization: clarifying complex interactions. Am J Pathol. 1998;153: 665–670. 9736014 10.1016/S0002-9440(10)65607-6 PMC1852998

[10] Qaum T, Xu Q, Joussen AM, Clemens MW, Qin W, Miyamoto K, et al VEGF-initiated blood-retinal barrier breakdown in early diabetes. Invest Ophthalmol Vis Sci. 2001;42: 2408–2413. 11527957

[11] Takeda M, Mori F, Yoshida A, Takamiya A, Nakagomi S, Sato E, et al Constitutive nitric oxide synthase is associated with retinal vascular permeability in early diabetic rats. Diabetologia. 200;44: 1043–1050. 11484083 10.1007/s001250100588

[12] Asnaghi V, Gerhardinger C, Hoehn T, Adeboje A, Lorenzi M. A role for the polyol pathway in the early neuroretinal apoptosis and glial changes induced by diabetes in the rat. Diabetes. 2003;52: 506–511. 12540628 10.2337/diabetes.52.2.506

[13] Martin PM, Roon P, Van Ells TK, Ganapathy V, Smith SB. Death of retinal neurons in streptozotocin induced diabetic mice. Invest Ophthalmol Vis Sci. 2004;45: 3330–3336. 15326158 10.1167/iovs.04-0247

[14] Tso MO, Cunha-Vaz JG, Shih CY, Jones CW. Clinicopathologic study of blood-retinal barrier in experimental diabetes mellitus. Arch Ophthalmol. 1980;98: 2032–2040. 7436840 10.1001/archopht.1980.01020040884020

[15] Vinores SA, Gadegbeku C, Campochiaro PA, Green WR. Immunohistochemical localization of blood-retinal barrier breakdown in human diabetics. Am J Pathol. 1989;134: 231–235. 2916645 PMC1879597

[16] Weinberger D, Fink-Cohen S, Gaton DD, Priel E, Yassur Y. Non-retinovascular leakage in diabetic maculopathy. Br J Ophthalmol. 1995;79: 728–731. 7547782 10.1136/bjo.79.8.728 PMC505232

[17] Xu HZ, Le YZ. Significance of outer blood-retina barrier breakdown in diabetes and ischemia.Invest Ophthalmol Vis Sci. 2011;52: 2160–2164. 10.1167/iovs.10-6518 21178141 PMC3080181

[18] Dahrouj M, Desjardins DM, Liu Y, Crosson CE, Ablonczy Z. Receptor mediated disruption of retinal pigment epithelium function in acute glycated-albumin exposure. Exp Eye Res. 2015;137: 50–56. 10.1016/j.exer.2015.06.004 26070987 PMC4523492

[19] Garcia-Ramírez M, Hernández C, Palomer X, Vázquez-Carrera M, Simó R. Fenofibrate prevents the disruption of the outer blood retinal barrier through downregulation of NF-κB activity. Acta Diabetol. 2016;53: 109–118. 10.1007/s00592-015-0759-3 25936740

[20] Wang S, Du S, Wu Q, Hu J, Li T. Decorin prevents retinal pigment epithelial barrier breakdown under diabetic conditions by suppressing p38 MAPK activation. Invest Ophthalmol Vis Sci. 2015;56: 2971–2979. 10.1167/iovs.14-15874 25829413

[21] Jackson TL, Paraskevopoulos T, Georgalas I. Systematic review of 342 cases of endogenous bacterial endophthalmitis. Surv Ophthalmol. 2014;59: 627–635. 10.1016/j.survophthal.2014.06.002 25113611

[22] Ho V, Ho LY, Ranchod TM, Drenser KA, Williams GA, Garretson BR. Endogenous methicillin-resistant *Staphylococcus aureus* endophthalmitis. Retina. 2011;31: 596–601. 10.1097/IAE.0b013e3181ecccf0 21343874

[23] Durand ML. Endophthalmitis. Clin Microbiol Infect. 2013;19: 227–234. 10.1111/1469-0691.12118 23438028 PMC3638360

[24] Arevalo J, Jap A, Chee S, Zeballos D. (2010). Endogenous endophthalmitis in the developing world. Int Ophthalmol Clin. 2010;50: 173–187. 10.1097/IIO.0b013e3181d26dfc 20375870

[25] Greenwald MJ, Wohl LG, Sell CH. Metastatic bacterial endophthalmitis: a contemporary reappraisal. Surv Ophthalmol. 1986;31: 81–101. 3541265 10.1016/0039-6257(86)90076-7

[26] Jackson TL, Eykyn SJ, Graham EM, Stanford MR. Endogenous bacterial endophthalmitis: a 17-year prospective series and review of 267 reported cases. Surv Ophthalmol. 2003;48: 403–423. 12850229 10.1016/s0039-6257(03)00054-7

[27] Okada AA, Johnson RP, Liles WC, D'Amico DJ, Baker AS. Endogenous bacterial endophthalmitis. Report of a ten-year retrospective study. Ophthalmology. 1994;101: 832–838. 8190467

[28] Coburn PS, Wiskur BJ, Christy E, Callegan MC. The diabetic ocular environment facilitates the development of endogenous bacterial endophthalmitis. Invest Ophthalmol Vis Sci. 2012;53: 7426–7431. 10.1167/iovs.12-10661 23036996 PMC3487485

[29] Coburn PS, Wiskur BJ, Astley RA, Callegan MC. Blood-retinal barrier compromise and endogenous *Staphylococcus aureus* endophthalmitis. Invest Ophthalmol Vis Sci. 2015;56: 7303–7311. 10.1167/iovs.15-17488 26559476 PMC4642607

[30] Lin J, Siu L, Fung C, Tsou H, Wang J, Chen C, et al Impaired phagocytosis of capsular serotypes K1 or K2 *Klebsiella pneumoniae* in type 2 diabetes mellitus patients with poor glycemic control. J Clin Endocrinol Metab. 2006;91: 3084–3087. 16720670 10.1210/jc.2005-2749

[31] Park S, Rich J, Hanses F, Lee J. Defects in innate immunity predispose C57BL/6J-Leprdb/Leprdb mice to infection by *Staphylococcus aureus*. Infect Immun. 2009;77: 1008–1014. 10.1128/IAI.00976-08 19103772 PMC2643648

[32] Wang J, Iacovelli J, Spencer C, Saint-Geniez M. Direct effect of sodium iodate on neurosensory retina. Invest Ophthalmol Vis Sci. 2014;55: 1941–1953. 10.1167/iovs.13-13075 24481259 PMC4049579

[33] Reagan A, Gu X, Hauck SM, Ash JD, Cao G, Thompson TC, et al Retinal Caveolin-1 Modulates Neuroprotective Signaling. Adv Exp Med Biol. 2016;854: 411–418. 10.1007/978-3-319-17121-0_54 26427439

[34] Moyer AL, Ramadan RT, Novosad BD, Astley R, Callegan MC. *Bacillus cereus*-induced permeability of the blood-ocular barrier during experimental endophthalmitis. Invest Ophthalmol Vis Sci. 2009;50: 3783–3793. 10.1167/iovs.08-3051 19264886 PMC2880527

[35] Cheung AL, Eberhardt KJ, Chung E, Yeaman MR, Sullam PM, Ramos M, Bayer AS. Diminished virulence of a sar-/agr- mutant of *Staphylococcus aureus* in the rabbit model of endocarditis. J Clin Invest. 1994; 94: 1815–1822. 7962526 10.1172/JCI117530 PMC294579

[36] Jung J, Lee J, Yu SN, Kim YK, Lee JY, Sung H, et al Ocular involvement of *Staphylococcus aureus* bacteremia: incidence and risk factors. Antimicrob Agents Chemother. 2016 1 11. pii: AAC.02651-15. [Epub ahead of print]. 10.1128/AAC.02651-15 PMC4808144 26824952

[37] Sheen TR, Ebrahimi CM, Hiemstra IH, Barlow SB, Peschel A, Doran KS. Penetration of the blood-brain barrier by *Staphylococcus aureu*s: contribution of membrane-anchored lipoteichoic acid. J Mol Med (Berl). 2010;88(6): 633–639. 20419283 10.1007/s00109-010-0630-5 PMC2893325

[38] Machalińska A, Lubiński W, Kłos P, Kawa M, Baumert B, Penkala K, et al Sodium iodate selectively injuries the posterior pole of the retina in a dose-dependent manner: morphological and electrophysiological study. Neurochem Res. 2010;35: 1819–1827. 10.1007/s11064-010-0248-6 20725778 PMC2957578

[39] Xia H, Krebs MP, Kaushal S, Scott EW. Enhanced retinal pigment epithelium regeneration after injury in MRL/MpJ mice. Exp Eye Res. 2011;93: 862–872. 10.1016/j.exer.2011.09.020 21989111 PMC3249660

[40] Pitkänen L, Ranta VP, Moilanen H, Urtti A. Permeability of retinal pigment epithelium: effects of permeant molecular weight and lipophilicity. Invest Ophthalmol Vis Sci. 2005;46: 641–646. 15671294 10.1167/iovs.04-1051

[41] Moyer AL1, Ramadan RT, Thurman J, Burroughs A, Callegan MC. *Bacillus cereus* induces permeability of an in vitro blood-retina barrier. Infect Immun. 2008;76:1358–1367. 10.1128/IAI.01330-07 18268029 PMC2292856

[42] Herbert S, Ziebandt AK, Ohlsen K, Schäfer T, Hecker M, Albrecht D, et al Repair of global regulators in *Staphylococcus aureus* 8325 and comparative analysis with other clinical isolates. Infect Immun. 2010;78: 2877–2889. 10.1128/IAI.00088-10 20212089 PMC2876537

[43] Plata K, Rosato A, Wegrzyn G. *Staphylococcus aureus* as an infectious agent: overview of biochemistry and molecular genetics of its pathogenicity. Acta Biochim Pol. 2009; 56:597–612. 20011685

[44] Wirtz C, Witte W, Wolz C, Goerke C. Transcription of the phage-encoded Panton-Valentine leukocidin of *Staphylococcus aureus* is dependent on the phage life-cycle and on the host background. Microbiology. 2009;155: 3491–3499. 10.1099/mic.0.032466-0 19661179

[45] Otto M. (2010). Basis of virulence in community-associated methicillin-resistant *Staphylococcus aureus*. Annu Rev Microbiol. 2010; 64: 143–162. 10.1146/annurev.micro.112408.134309 20825344

[46] Löffler B, Hussain M, Grundmeier M, Brück M, Holzinger D, Varga G, et al *Staphylococcus aureus* panton-valentine leukocidin is a very potent cytotoxic factor for human neutrophils. PLoS Pathog. 2010;6:e1000715 10.1371/journal.ppat.1000715 20072612 PMC2798753

[47] Ibberson CB, Jones CL, Singh S, Wise MC, Hart ME, Zurawski DV, et al *Staphylococcus aureus* hyaluronidase is a CodY-regulated virulence factor. Infect Immun. 2014; 82: 4253–4264. 10.1128/IAI.01710-14 25069977 PMC4187871

[48] Novick RP. Autoinduction and signal transduction in the regulation of staphylococcal virulence. Mol Microbiol. 2003; 48: 1429–1449. 12791129 10.1046/j.1365-2958.2003.03526.x

[49] Booth MC, Cheung AL, Hatter KL, Jett BD, Callegan MC, Gilmore MS. Staphylococcal accessory regulator (sar) in conjunction with agr contributes to *Staphylococcus aureus* virulence in endophthalmitis. Infect Immun. 1997;65: 1550–1556. 9119503 10.1128/iai.65.4.1550-1556.1997 PMC175169

[50] Booth MC, Hatter KL, Miller D, Davis J, Kowalski R, Parke DW, et al Molecular epidemiology of *Staphylococcus aureus* and *Enterococcus faecalis* in endophthalmitis. Infect Immun. 1998;66: 356–360. 9423880 10.1128/iai.66.1.356-360.1998 PMC107899

[51] Callegan M, Booth M, Jett B, Gilmore M. Pathogenesis of gram-positive bacterial endophthalmitis. Infect Immun. 1999; 67: 3348–3356. 10377112 10.1128/iai.67.7.3348-3356.1999 PMC116517

[52] Wiskur BJ, Hunt JJ, Callegan MC. Hypermucoviscosity as a virulence factor in experimental *Klebsiella pneumoniae* endophthalmitis. Invest Ophthalmol Vis Sci. 2008;49: 4931–4938. 10.1167/iovs.08-2276 18586871 PMC2576484

[53] Novick R. Properties of a cryptic high-frequency transducing phage in *Staphylococcus aureus*. Virology. 1967;33: 155–166. 4227577 10.1016/0042-6822(67)90105-5

[54] Ramadan RT, Ramirez R, Novosad BD, Callegan MC. Acute inflammation and loss of retinal architecture and function during experimental *Bacillus* endophthalmitis. Curr Eye Res. 2006;31: 955–965. 17114121 10.1080/02713680600976925

[55] Ramadan RT, Moyer AL, Callegan MC. A role for tumor necrosis factor-alpha in experimental *Bacillus cereus* endophthalmitis pathogenesis. Invest Ophthalmol Vis Sci. 2008;49:4482–4489. 10.1167/iovs.08-2085 18586878 PMC2574773

[56] Bæk KT, Frees D, Renzoni A, et al Genetic Variation in the *Staphylococcus aureus* 8325 Strain Lineage Revealed by Whole-Genome Sequencing. Otto M, ed. PLoS ONE. 2013;8: e77122 10.1371/journal.pone.0077122 24098817 PMC3786944

[57] Rasband WS, ImageJ, U. S. National Institutes of Health, Bethesda, Maryland, USA, http://imagej.nih.gov/ij/, 1997–2014.

[58] Schneider CA, Rasband WS, Eliceiri KW. NIH Image to ImageJ: 25 years of image analysis. Nature Methods. 2012;9: 671–675. 22930834 10.1038/nmeth.2089 PMC5554542

[59] Abramoff MD, Magalhaes PJ, Ram SJ. Image Processing with ImageJ. Biophotonics International. 2004;11: 36–42.

